# Straight stem and threaded cup in patients under 60 years of age: 28.8–30.2 years of follow-up

**DOI:** 10.1186/s13018-020-02102-w

**Published:** 2020-11-26

**Authors:** L. Pisecky, J. Allerstorfer, B. Schauer, G. Hipmair, R. Hochgatterer, N. Böhler, T. Gotterbarm, M. C. Klotz

**Affiliations:** 1 Department for Orthopedics and Traumatology, Kepler University Hospital GmbH, Krankenhausstraße 9, 4020 Linz, Austria; 2 Johannes Kepler University Linz, Altenberger Strasse 96, 4040 Linz, Austria

**Keywords:** Total hip arthroplasty, Threaded cup CSF, Straight stem Alloclassic SL, Long-term follow-up, Young patients

## Abstract

**Purpose:**

The aim of this retrospective observational study of one cohort was to evaluate the long-term outcome in patients younger than 60 years after total hip arthroplasty using a straight uncemented stem and an uncemented threaded cup.

**Methods:**

Between 1986 and 1987, 75 hips of 75 patients (mean age, 53.35 ± 6.17 years) were consecutively implanted with an Alloclassic Zweymüller/Alloclassic SL stem and an Alloclassic CSF cup. Forty-four patients had died over the last 30 years. The remaining 31 patients (mean age, 82.9 ± 6.4 years) were reinvited for follow-up examinations. Clinical and radiographic evaluations were carried out.

**Results:**

At a mean follow-up of 29.5 (28.8–30.2), 4 patients (5.3%) were lost to follow-up.

For the endpoint aseptic loosening (defined as the removal of stem or the cup for 2 cases), the overall survival rate is 97.3%. For the endpoint revision for any reason (22 patients), the survival rate is 70.6%. Eleven patients needed an exchange of head and liner, caused by wear. The average time from implantation until change of head and liner was 21.44 years (SD 5.92). Other reasons for revision surgery were septic loosening (3 cases), aseptic loosening of stem and cup (1 case), aseptic loosening of stem (1 case), periprosthetic calcification (2 cases), implant fracture (1 case), periprosthetic fracture (1 case), intraoperative fissure of stem (1 case), and total wear of liner including cup (1 case).

**Conclusion:**

The combination of a straight stem (Alloclassic) and a screw cup (CSF) shows excellent results in young patients under the age of 60 at ultra-long-term follow-up at 30 years. Revisions due to wear of the polyethylene liner are more likely than in the older patients.

## Introduction

Various studies and registry data deal with short- and midterm results of the cementless implanted Alloclassic Zweymüller hochgezogen, the Alloclassic SL, and the Alloclassic CSF screw cup system [1–3]. These implants have excellent results in older patients [3]. Some studies describe long-term results up to 20 years [4]. Other well-established stem systems are reported to have excellent long-term results such as Bicontact [5, 6], Taperloc [7], Corail [8, 9], and CLS [10–12] as well as threaded cups such as T-TAP ST [13].

Recent trends lead to bone and muscle preserving implants such as spherical press-fit cups and bone preserving, metaphyseal fixed short stems, especially in younger and more active patients. According to some authors, available data does not show satisfying evidence regarding the benchmark of 90% survivorship after 10 years, set by the National Institute of Clinical Excellence, NICE [14]. Up to now, only straight stems have an ODEP 13A* rating (http://www.odep.org.uk/products.aspx [15]).

Threaded cups and conventional straights stems have proven excellent overall long-term results in various cohorts.

Nevertheless, there is only limited published data for the combination of a straight stem with a screw cup in younger patients, who are mostly more physically active and demanding

We already published first results of our cohort 15 years ago with excellent outcome [3]. Up to now, there is no published data reporting clinical and radiological results for a screw cup in combination with a straight stem in patients less than 60 years of age at time of implantation with an ultra-long-term follow-up of up to 30 years.

## Materials and methods

We established a retrospective observational study of one cohort. Between 1986 and 1987 seventy-five hips in 75 consecutive patients 60 years of age or younger at time of surgery were treated in our institution with a cementless total hip arthroplasty system. All patients received a straight uncemented stem (*n* = 46, Alloclassic Zweymueller/*n* = 29, Alloclassic SL) and a uncemented threaded cup (*n* = 75; Alloclassic CSF (AlloPro/Sulzer Medica; Centerpulse; Winterthur, Switzerland)). In all cases, a standard non-highly cross-linked PE liner was used with 28-mm ceramic heads with 12/14 trunnion (Cerasul, AlloPro/SulzerMedica). The group included 38 women and 37 men. Mean age at surgery was 53.35 years (32.5–60.5; standard deviation 6.17; 95% confidence interval 51.9–54.7).

Forty-four patients (44 hips) had died over the past 30 years. Using data from a previous study, we know for 42 of them if the arthroplasty system was in situ at death or revised for any reason.

**Fig. 1:**
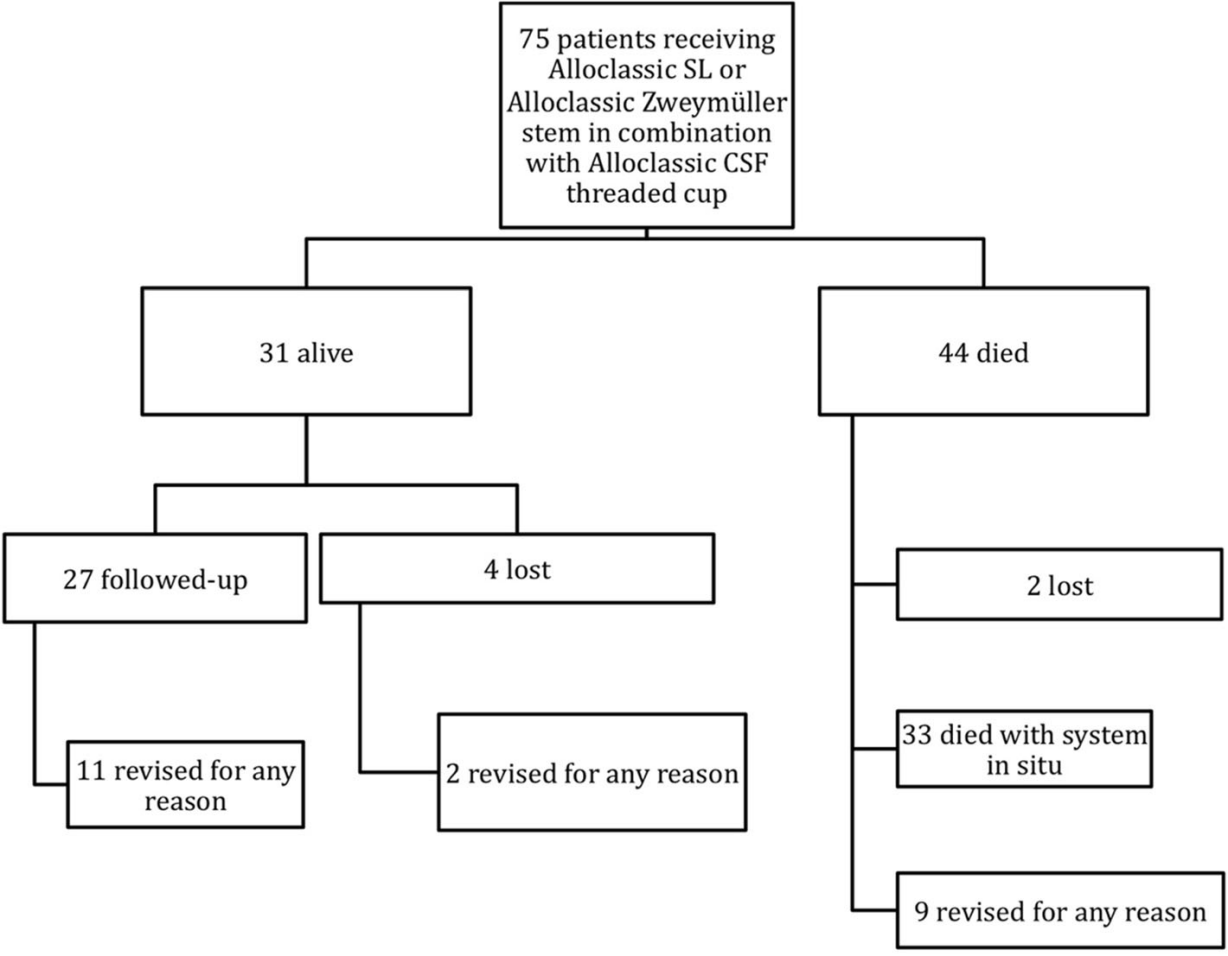
Algorithm leading to revision surgery for any reason

Four patients being still alive were lost to follow-up. The remaining 27 (Fig. 1) patients (12 male, 15 female; 27 hips) were available and had a mean age of 82.9 years at follow-up (72.4–90.5; SD 6.4; 95% CI 80.5–85.5).

The patients were interviewed by telephone if the prostheses were still in situ and if there was any revision surgery or any problems over the past years, concerning the prostheses. If feasible, the patients were invited to come to our outpatient clinic for a routine clinical and radiological check-up. We were able to assess 16 patients clinically and radiologically. All other 11 patients refused to come to the hospital but data from a structured telephone-interview was available.

The surgical indication (Table 1) for total hip arthroplasty was primary osteoarthritis in 55 cases (73%) and secondary osteoarthritis in 8 cases (16%). Others were revision surgeries in 12 cases (10.6%).

**Table 1:** Indications for implantation of the cementless Alloclassic/CSF THA-system

Indication	Absolute number	Percentage
Primary osteoarthritis	55	73.3
Necrosis of the femoral head	7	9.3
Dysplasia	5	6.7
St.p. loosening of cup	5	6.7
St.p. Girdlestone procedure	3	4
Trauma	1	1.3
Perthes	1	1.3

For analyzing the osteolytic zones on biplanar x-rays, the image editing software GIMP, version 2.8, was used. In detail, using histograms made it possible to analyze the amount of colored pixels which was needed to cover the ball head and the radiolucent zones. With these measurements, the size of the osteolysis could be calculated. The technique was described in 2017 by the authors [16].

We calculated the survival rate of the implanted system including graphs for survival curves according to Kaplan-Meier, the adverse events correlating with the implants over the past 30 years, the occurrence of radiolucent lines and osteolytic zones in the recent weight bearing x-rays according to the classification of DeLee and Charnley [17] and Gruen et al. [18] on the a-p view, and the patients’ satisfaction with the result after 30 years. Confidence intervals are given for calculated values, if applicable. Patients without follow-up were excluded from further analysis.

Survival curves were calculated for all implanted stems for aseptic loosening as well as revision for any reason. The software used was Microsoft Excel 12.2.6 and jamovi 0.9.5.12 for detailed statistical analysis.

## Results

### Survival rate and adverse events (Table 2)

We had two cases of aseptic loosening as well as three cases of septic loosening.

**Table 2:** Reasons for revision surgery

Type of failure	Absolute number	Absolute risk
Wear	11	14.67%
Septic loosening	3	4%
Aseptic loosening of stem	2	2.67%
Periprosthetic fracture	2	2.67%
Aseptic loosening of cup	2; 1 with loose stem; 1 with worn-out liner	2.67%
Major calcification	2	2.67%
Implant fracture (stem)	1	1.33%
Totally worn-out liner	1	1.33%
Overall patients needing revision surgery	22	29.3%

**Fig. 2:**
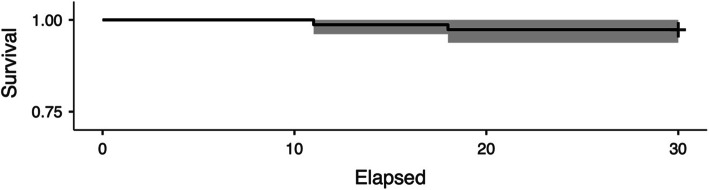
Kaplan-Meier survival curve with 95% CI for the endpoint “aseptic loosening of the stem or cup”

**Fig. 3:**
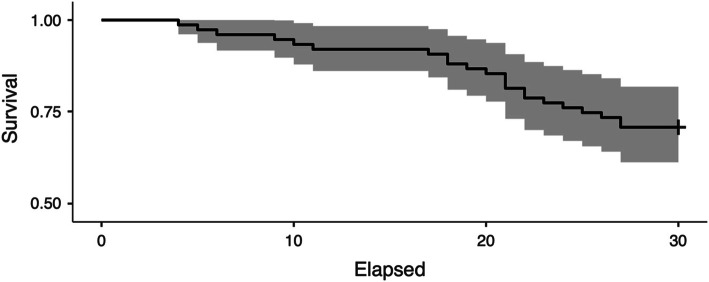
Kaplan-Meier survival curve with 95% CI for the endpoint “revision for any reason”

If the endpoint is defined as the removal of the stem or cup for aseptic loosening (2 cases), the overall calculated survival rate is 97.33% (Fig. 2). If the endpoint is revision for any reason (22 cases), the calculated survival rate is 70.6% (Fig. 3). Eleven patients needed revision surgery for exchange of head and liner; one of them was operated twice on one hip due to wear. The mean time from implantation until change of head and liner was 21.44 years (SD 5.92).

**Fig. 4:**
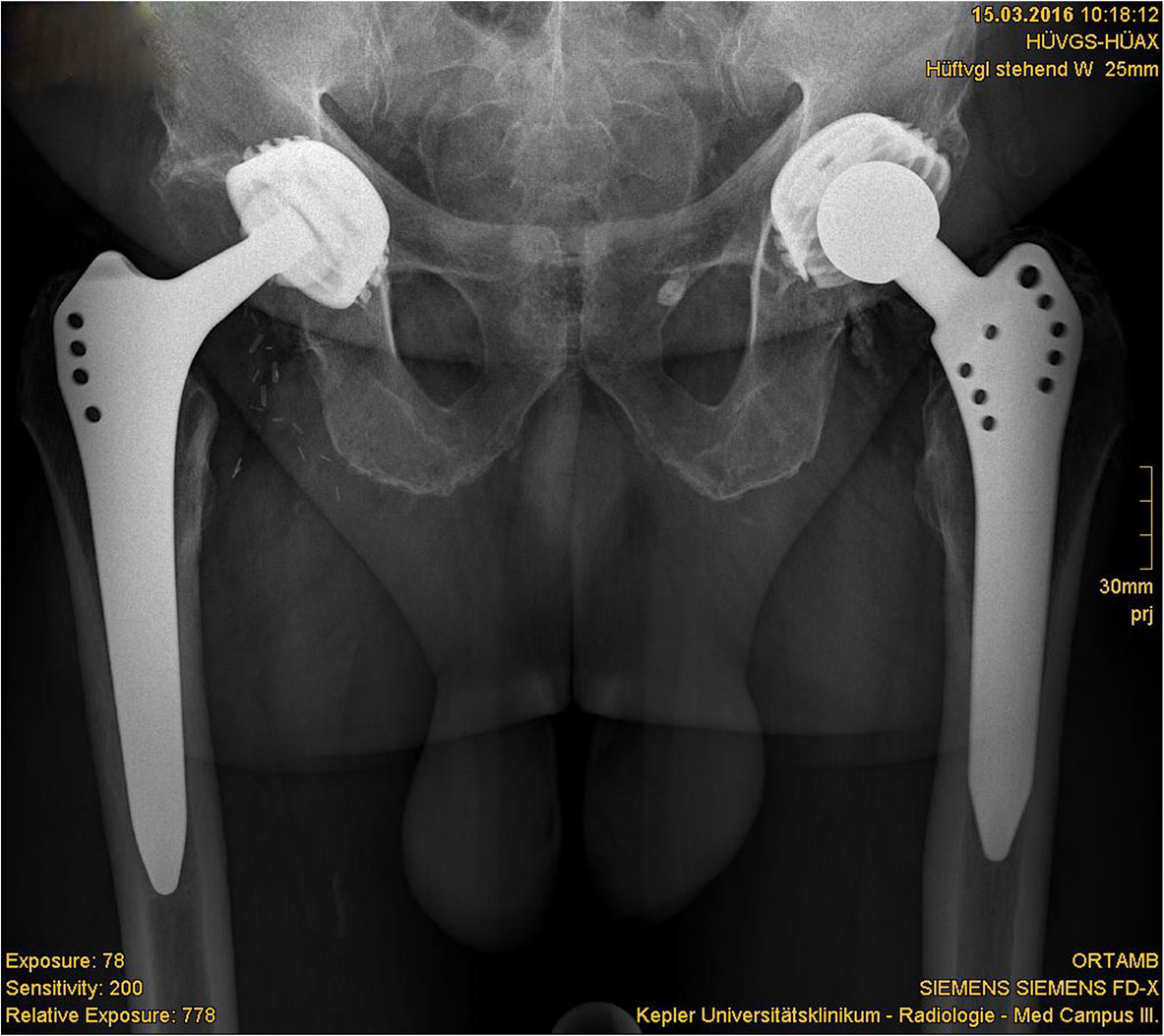
Radiograph showing excellent results after 30 years in a highly active 73-year-old male (l)

**Fig. 5:**
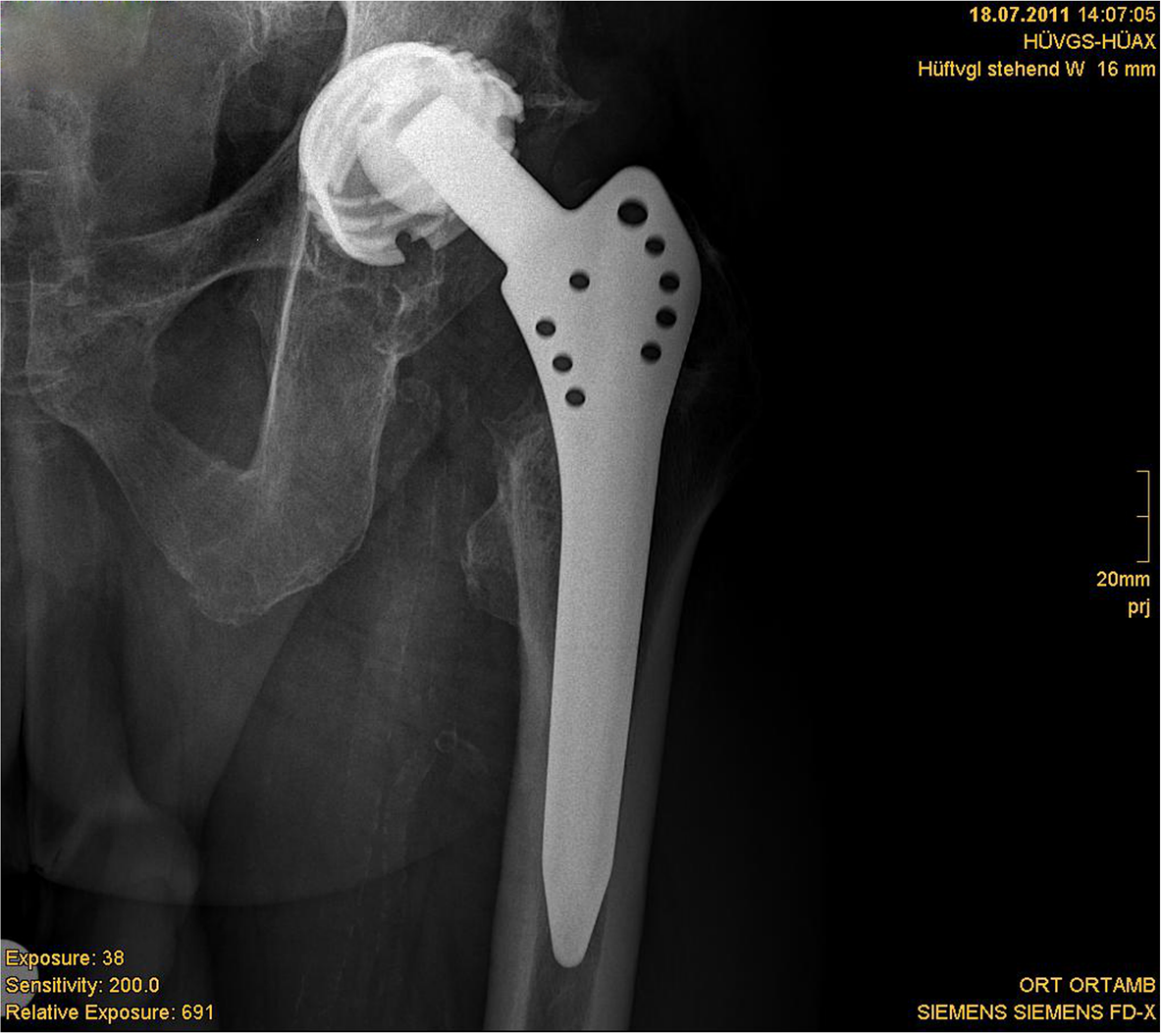
Radiograph showing totally worn-out liner after 25 years

**Fig. 6:**
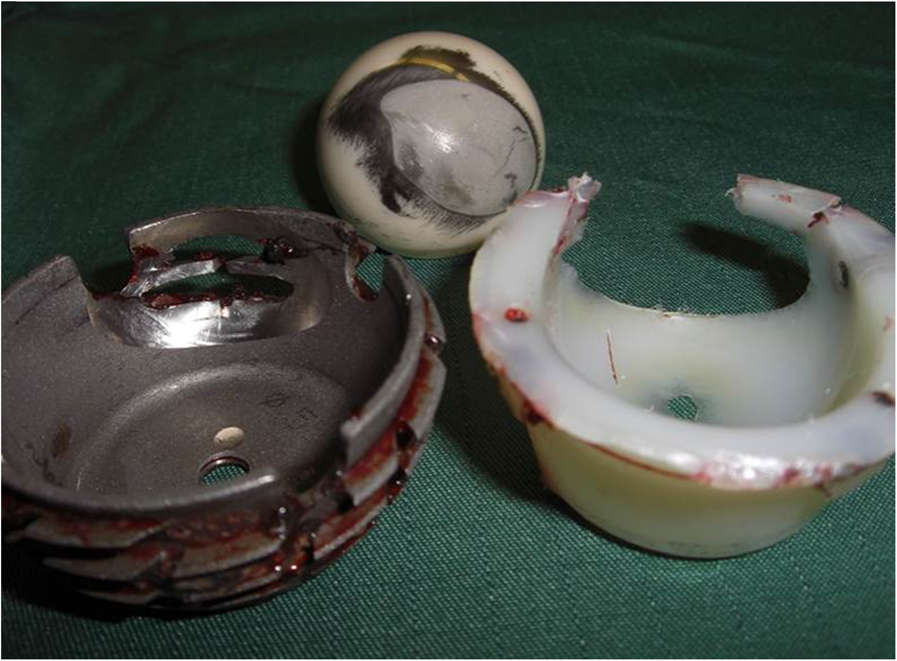
Intraoperative picture showing totally worn-out liner and affected cup

**Fig. 7:**
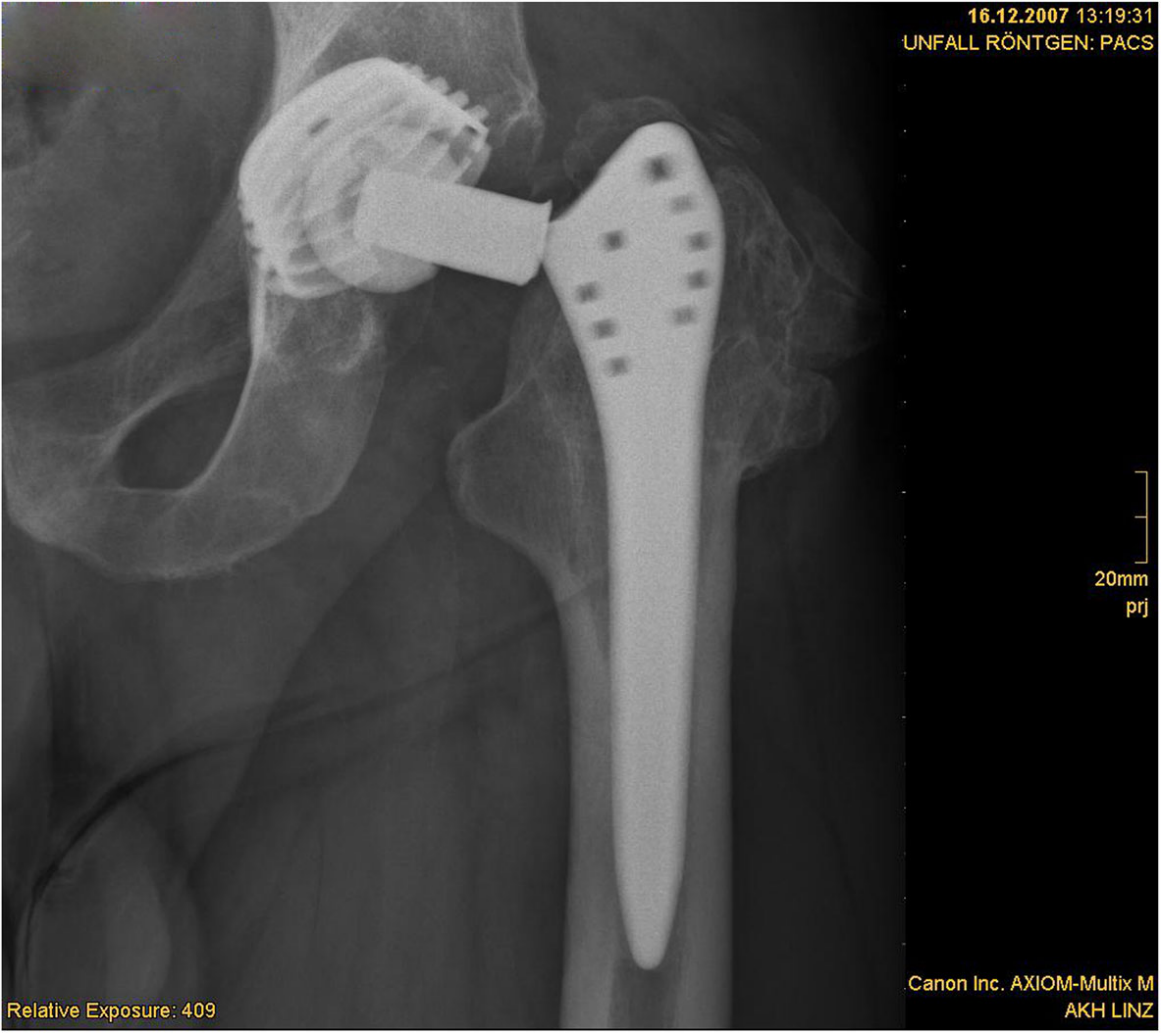
Radiograph showing implant fracture after 20 years

One totally worn-out liner led to the revision surgery of the acetabular component due to destruction of the acetabular component (Figs. 4, 5, 6, and 7).

Those patients, who died over the last 30 years, have lived with the arthroplasty system for an average of 17.68 years (3.7–28.3; SD 7.3; 95% CI 15.9–19.9).

### Radiological

According to the biplanar measurement of the x-rays using the technique as described by the study group in 2017, most of the radiolucent lines and osteolytic zones were found in the proximal Gruen-zones 1 (19 hips) and 7 (5 hips), in only 2 hips in zone 6. Osteolytic zones around the cup were seen in the lateral DeLee and Charnley zone 1 in 8 hips.

Being aware of the mode of stress-shielding around components, none of the implanted stems and cups was considered to be at risk.

### Clinical

Overall satisfaction of our patients with the implanted total hip arthroplasty system was very good (Fig. 4). None of the 27 patients described pain.

## Discussion

From 1986 on, our institution has been implanting the described Alloclassic SL and CSF screw cup total hip arthroplasty system in primary and secondary hip surgery.

This is the first study to provide a 30-year follow-up for young patients under 60 years of age treated with an uncemented total hip arthroplasty system consisting of a porous-coated straight stem and threaded.

Pursuing our published data for the Alloclassic stem system after 30 years of follow-up, our evaluation in combination with the cementless implanted Alloclassic CSF cup system is the first ultra-long-term study in young and demanding patients with a mean follow-up of 29.5 years [19, 20]. During this timespan, a broad variety of complications occurred in our cohort. Reasons for revisions were various and represented the known modes of failure of total hip arthroplasty systems.

Nevertheless, for a majority of 70.6%, the implantation of the evaluated THA system was the last surgery needed involving the replaced joint.

Previous data shows that youth as well as higher activity levels may increase the incidence of mechanical failure of total hip prostheses. Wangen et al. showed a failure rate of 49% for the acetabular components (press-fit and screw cups) due to mechanical failure in patients younger than 30 years after a mean follow-up of 16 years [21]. The main type of failure in our study did not occur in the interface between cup and bone, but wear of the polyethylene liner was the main reason for revision.

Reigstad et al. presented a long-term follow-up (15–18 years) for a tapered stem (Zweymüller SL) and a threaded cup (Endler) in 75 hips. Averaging 52 years of age at surgery, the cohort is comparable to the one of this study group. The survival analysis for the implanted system showed a Kaplan-Meier survival rate of 88%, whereof 6 cups had to be revised due to polyethylene wear. The prefixed polyethylene was stated to be a serious drawback, because in case of progression of wear, the explantation of the whole cup system is required [22].

In our population group, 29.6% needed revision surgery for any reason, which seems to be on a high level compared to other published cohorts with a long-term follow-up in the third decade. It has to be noted that in those cases, which needed revision surgery to exchange head and liner, a regular PE liner had been implanted in primary surgery. As soon as ultra-high cross-linked UHMWPE liners have entered the market in 1998, secondary surgery was performed using this long material, which has proven about 10 times less wear in long-term follow-up cohorts. As a result, a far smaller revision rate due to wear can be expected [23–25].

It can be assumed that the known cost-effectiveness of THA gets stronger when the revision rate decreases [26, 27].

Regarding the low wear rates of modern materials, the fear of wear-associated complications in younger individual over decades does not seem to be justified.

Most of the radiolucent lines and osteolytic zones were seen in the lateral zone I according to DeLee and Charnley describing changes at the cup. Radiolucent lines around the stem were commonly seen in Gruen zones 1 and 7, but none of the implanted systems was considered to be at risk [3, 28]. The authors were able to show in a previous study that there may be a positive effect for the reduction of osteolytic zones around the stem caused by wear when performing an exchange of head and liner [16].

The results of this study group are comparable to previous published cohorts as shown by Delaunay, giving survival rates of 99% for aseptic loosening after a timespan of 7 to 8 years [1].

The study group of Pieringer et al. presented 98.4% surviving cups after 157 months for the endpoint of aseptic loosening in 2006 [29]. The cohort of Busch et al. gave results of 89% survival rate (revision for any reason; including cup and stem) after 17 years in 2012 [4]. Schröder et al. gave a report on their cohort of revision cases with a survival rate of 95% for the cup after 6.1 years [2]. Within the Czech arthroplasty register, 2677 evaluated stems showed a survival rate of 99.81% after 1 to 11 years [30].

Other stems with excellent long-term results in younger patients such as CLS Spotorno (72.8 any reason after 23.8 years) [10–12], Bicontact (93.57% at 12 years) [5, 6], and Corail (95% at 12 years) [8, 9] show good results up to 24 years of follow-up, but none of the mentioned cohorts give results for a period up to the end of the third decade as we do. Published data does sometimes not focus on the long-term problems such as wear of early polyethylene liners; therefore, the reason for aseptic loosenings stays unclear in most cases. We observed a high number of worn-out liners, so we assume that the reason for our aseptic loosenings may be the result of wear after a long timespan.

It has to be stated that the criterion “revision for any reason” increases with the years and usage because of progressive wear and as consequence the need for replacing head and liner [31]. Comparison with data from our previous publications shows that all of the patients, who needed an exchange of head and liner, were not older than 60 years at primary surgery.

Knowing the excellent results for aseptic loosening of the discussed system, a successing arthroplasty system needs further benchmarks to prove superiority. Criteria such as bone loss in revision surgery and reproducibility of surgical results are arguments to pursue the development and evaluation of bone sparing, and more anatomically shaped arthroplasty systems [32–34].

Our evaluation is limited by the small number of patients being alive after 30 years. Furthermore, most of the surviving patients are in a bad general medical condition or have moved far away from our institution, which leads to a very small number of clinical evaluations. Nevertheless, our primary aim was not the collection of clinical scores, but the survival rate of the implant and the resulting complications during a 30-year timespan in younger, more demanding patients. This objective was achieved satisfactorily.

The evaluated system is very reliable in primary and secondary THA, as shown in previous studies as well as in this one. In our institution, it has been the basic implant for many years. It has been our backup-system in cases of failure of anatomically shaped stems and spherical press-fit cups for a long period of time.

## Conclusion

The current study is the first one to show a 30-year follow-up for the described straight stem in combination with the threaded cup in patients younger than 60 years of age at implantation. The combination of the discussed total hip arthroplasty system shows excellent results in young patients under the age of 60 at ultra-long-term follow-up at 30 years and sets the benchmark for successing total hip arthroplasty systems such as anatomically shaped stems and spherical cups. Revisions due to wear of the polyethylene liner are more likely than in the older patients.

## Data Availability

Access to published data will be granted on reasonable request.

## Abbreviations

CI: Confidence interval
CLS: Cementless stem
CSF: Conical self-tapping fixation
NICE: National Institute of Clinical Excellence
ODEP: Orthopaedic Data Evaluation Panel
PE: Polyethylene
SD: Standard deviation
SL: Stepless
THA: Total hip arthroplasty
T-TAP: Threaded hemispheric titanium shell
UHMWPE: Ultra high molecular weight polyethylene
